# Opposite regulation of piRNAs, rRNAs and miRNAs in the blood after subarachnoid hemorrhage

**DOI:** 10.1007/s00109-020-01922-x

**Published:** 2020-05-18

**Authors:** Rafal Morga, Malgorzata Borczyk, Michal Korostynski, Marcin Piechota, Dzesika Hoinkis, Slawomir Golda, Tomasz Dziedzic, Agnieszka Slowik, Marek Moskala, Joanna Pera

**Affiliations:** 1 Department of Neurosurgery and Neurotraumatology, Faculty of Medicine, Jagiellonian University Medical College, Krakow, Poland; 2 Department of Molecular Neuropharmacology, Maj Institute of Pharmacology, Polish Academy of Sciences, Krakow, Poland; 3 Intelliseq sp. z o.o., Krakow, Poland; 4 Department of Neurology, Faculty of Medicine, Jagiellonian University Medical College, ul. Botaniczna 3, 31-503 Krakow, Poland

**Keywords:** Subarachnoid hemorrhage, Intracranial aneurysm, Small noncoding RNAs, Peripheral blood, RNA sequencing

## Abstract

**Abstract:**

Multiple classes of small RNAs (sRNAs) are expressed in the blood and are involved in the regulation of pivotal cellular processes. We aimed to elucidate the expression patterns and functional roles of sRNAs in the systemic response to intracranial aneurysm (IA) rupture. We used next-generation sequencing to analyze the expression of sRNAs in patients in the acute phase of IA rupture (first 72 h), in the chronic phase (3–15 months), and controls. The patterns of alterations in sRNA expression were analyzed in the context of clinically relevant information regarding the biological consequences of IA rupture. We identified 542 differentially expressed sRNAs (108 piRNAs, 99 rRNAs, 90 miRNAs, 43 scRNAs, 36 tRNAs, and 32 snoRNAs) among the studied groups with notable differences in upregulated and downregulated sRNAs between the groups and sRNAs categories. piRNAs and rRNAs showed a substantial decrease in RNA abundance that was sustained after IA rupture, whereas miRNAs were largely upregulated. Downregulated sRNA genes included piR-31080, piR-57947, 5S rRNA, LSU-rRNA, and SSU-rRNA s. Remarkable enrichment in the representation of transcription factor binding sites was revealed in genomic locations of the regulated sRNA. We found strong overrepresentation of glucocorticoid receptor, retinoid x receptor alpha, and estrogen receptor alpha binding sites at the locations of downregulated piRNAs, tRNAs, and rRNAs. This report, although preliminary and largely proof-of-concept, is the first to describe alterations in sRNAs abundance levels in response to IA rupture in humans. The obtained results indicate novel mechanisms that may constitute another level of control of the inflammatory response.

**Key messages:**

- A total of 542 sRNAs were differentially expressed after aneurysmal SAH comparing with controls
- piRNAs and rRNAs were upregulated and miRNAs were downregulated after IA rupture
- The regulated sRNA showed an enrichment in the representation of some transcription factor binding sites
- piRNAs, tRNAs, and rRNAs showed an overrepresentation for GR, RXRA, and ERALPHA binding sites

**Electronic supplementary material:**

The online version of this article (10.1007/s00109-020-01922-x) contains supplementary material, which is available to authorized users.

## Introduction

Intracranial aneurysm (IA) rupture results in many systemic effects and strongly influences immune system function. The molecular mechanisms driving the effects of subarachnoid hemorrhage (SAH) remain to be elucidated to improve the management of patients with ruptured IAs. One of the elements of the systemic effects of IA rupture is changes in the transcriptome of peripheral blood cells. To date, protein-coding mRNAs are the RNA class to have been investigated first and most intensively using microarrays initially and then next-generation sequencing techniques [1–4]. However, there is an increasing body of evidence indicating that noncoding RNAs (ncRNAs) play a crucial role in many pathological conditions. According to their length, ncRNAs are divided into two large categories: long (> 200 bp) and small (< 200 bp) ncRNAs. Small ncRNAs (sncRNAs) are a highly heterogenic group comprising microRNAs (miRNAs), piwi-interacting RNAs (piRNAs), short interfering RNAs (siRNAs), small nucleolar RNAs (snoRNAs), small nuclear RNAs (snRNAs), promoter-associated small RNAs (PASRs), transfer RNAs (tRNAs), and ribosomal RNAs (rRNAs). In general, these RNAs are regulatory molecules influencing gene transcription, translation, genome integrity preservation, and epigenetic regulation [5–8].

In our previous studies, we focused on the effects exerted by IA rupture on the expression of mRNAs and miRNAs in peripheral blood cells [4, 9]. In this preliminary study, we investigated changes in the expression of small ncRNAs other than miRNAs in peripheral blood cells using deep transcriptome sequencing in a single cohort of patients. To assess time-related changes, patients in the acute (days) and chronic (months) phases of aSAH were included in the study.

## Materials and methods

### Patients

The studied cohort was described elsewhere [4]. Briefly, patients with aneurysmal SAH were prospectively recruited from consecutive patients of the Departments of Neurology or Neurosurgery and Neurotraumatology, University Hospital, Krakow. Two independent patient groups were analyzed: acute (within the first 72 h after IA rupture) and chronic (within 3–15 months after SAH). Control subjects (C) were recruited from patients in the Department of Neurology who suffered from headaches. Demographic and risk factor data were collected using a dedicated questionnaire.

All subjects were Caucasian. Written informed consent was obtained from all participants (or guardians of participants) before inclusion in the study. The local ethics committee approved the study.

### Blood collection and RNA extraction

Venous whole blood was collected before neurosurgical interventions in PAXgene Blood RNA Tubes (PreAnalytiX, GmbH, Switzerland), mixed, and kept at room temperature for at least 2 h. The tubes were subsequently frozen and stored at – 70 °C until further processing. Total RNA was purified from blood samples using the PAXgene Blood RNA Kit (PreAnalytiX) according to the manufacturer’s protocol and was treated with DNase. RNA concentrations were measured using a NanoDrop ND-1000 Spectrophotometer (NanoDrop Technologies, Montchanin, DE), and RNA quality was determined by ChIP-based capillary electrophoresis utilizing the Agilent RNA 6000 Nano Kit and an Agilent Bioanalyzer 2100 (Agilent, Palo Alto, CA) according to the manufacturer's protocols.

### Small RNA sequencing

sRNA library preparation and sequencing were performed with Illumina sequencing technology. The sRNA library was generated with the TruSeq Small RNA Library Kit. Briefly, 3′ and 5′ adapters were ligated into 1 μg of total RNA with T4 RNA ligase. Then, reverse transcription was performed with the Illumina sRNA RT-Primer, and cDNA was amplified by PCR (11 cycles) using the Illumina small RNA primer set. The amplified total cDNA library was purified and size selected (insert size 22–30 bp) in 6% Novex TBE gel. The transcriptome libraries were sequenced on a HiSeq2500™ (Illumina) with the following parameters: SE50 (single end) and 10 M clean reads, which yielded a minimum of 500 Mb of raw data per sample (SE50, 10 M, 500 Mb). The sRNA-seq data were submitted to the NCBI Sequence Read Archive (SRA): SRP150595. The detailed analysis of the regulated miRNA has been published elsewhere [9]. In this study, we focus on multiple different classes of sRNAs that are differentially expressed in the blood in response to IA rupture. We reanalyzed profiles of miRNA regulation to compare and contrast with the other classes.

### sRNA data analysis

The sequence read quality was evaluated using the FastQC (0.11.5) quality filter module. The raw reads were mapped to the reference human genome hg38 using BWA (version 0.7.17). The expression levels were quantified using IntersectBed (v2.28.0) and GTF with data from the DASHR database of human small noncoding RNAs in human tissues and cell types (v2.0). sRNA transcripts with counts per million (CPM) values above 2 in at least 10 samples were considered detectable and were used for further analysis.

### Identification of differentially expressed sRNA

All statistical analyses were performed using R software v3.4.3. A normalization step was performed because the total number of reads from different experiments was not the same, and variations in the number of reads of individual sRNA may be attributable to sequencing depth. The total clone count was the sum of frequencies of all the residual unique sequences after filtering. Differential expression was analyzed with the quasi-likelihood F-test from the edgeR package (v.3.6.8). The false discovery rate (FDR) was estimated using the Benjamini-Hochberg method. Sequences with FDR < 0.1 were considered to be differentially expressed. Statistical power of the analysis was estimated with two methods: (1) according to Hart et al. [10] with depth = 676, cv = 0.47 and was 33.6% for 1.2 fold-change (FC), 85% for 1.5 FC, 97.2% for 1.7 FC, and 99.86% for 2 FC; (2) by shuffling sRNA counts, simulating a given FC in a subset of the dataset and detecting the number of changed sequences that passed the 0.1 FDR. Here, 99.4% of sequences were detected at simulated 1.2 FC in 10% of dataset. Sequences with fold-change > 1.2 compared with controls (C) were considered regulated in particular conditions. After the fold-change filter was applied, 516 sequences remained.

### Conservation analysis

For conservation analysis, phastCons scores for multiple alignments of 99 vertebrate genomes (list: http://hgdownload.cse.ucsc.edu/goldenpath/hg38/phastCons100way/) to the human genome were averaged across each sRNA sequence (noncovered bases were given a score of 0). Conservation scores have values between 0 and 1 (1: means that all bases are conserved in all 100 genomes).

### Overrepresentation of ChIP-seq-defined DNA features

To investigate the biological mechanisms involved in the control of sRNA expression after IA rupture, we searched for overrepresented transcription factor binding sites on genomic regions of the regulated transcripts. We employed available ChIP-seq data (Consortium, 2012) using the online resource seqinspector (seqinspector.cremag.org). The batch coordinate conversion (liftOver) was used to convert genome coordinates between hg38 and hg19 assemblies (516 regulated sRNAs were converted to 508). The set of genomic coordinates from sRNA-seq was used as an input. The background reference set was defined as 455 randomly selected, nonregulated sRNAs with mean expression levels in the blood similar to those of the regulated sRNAs. The difference between the query and reference datasets was calculated by *t* test with the use of Bonferroni correction. Where applied, the difference between class composition was analyzed using the χ2 test.

## Results

### Alterations in sRNA abundance levels in response to IA rupture

The study comprised 19 patients in the acute phase of IA rupture (RAA), 20 patients in the chronic phase of SAH (RAC), and 20 control subjects (C) (Table 1). The clinical characteristics of the patients are published elsewhere [4].

**Table 1:** Baseline characteristics of the patients

	RAA (*n* = 19)	RAC (*n* = 20)	C (*n* = 20)
Age, years (median, IQR)	54 (48–62)	50 (41–56)	55 (50–60)
Female, %	73.7	90.0	55.0*
Hypertension, %	57.9	55.0	50.0
Smoking, %	31.6	40.0	30.0
Excessive drinking, %	0	5.0	10.0
Diabetes mellitus, %	5.3	5.0	10.0
Hyperlipidemia, %	5.3	0	15.0
Admission Hunt-Hess score (median, IQR)	2 (1–3)		
Aneurysms location			
Anterior circulation, *n*	17	16	
Posterior circulation, *n*	2	4	

*RAA*, acute phase of intracranial aneurysm (IA) rupture; *RAC*, chronic phase of IA rupture; *C*, control subjects; *IQR*, interquartile range; *GCS*, Glasgow Coma Scale

*p < 0.05 RAC vs. C

We used NGS to comprehensively examine the expression levels of sRNAs derived from the peripheral blood. Normalized miRNA abundance levels (CPM) were measured for the small transcripts annotated in the GRCm38.p13 genome release. A total of 1766 genes were detected at the threshold of CPM > 2 in at least 10 samples. To annotate the genome-mapped sRNA reads, we referred to the UCSC Genome Browser and Ensembl database. For differential expression detection, results were considered significant if their FDR was < 0.01. Comparing RAA, RAC, and controls, we found 542 differentially expressed sRNAs that included 516 sRNAs with an abundance change of more than 1.2-fold compared with the control. This list of 516 genes was used for further analysis.

**Fig. 1:**
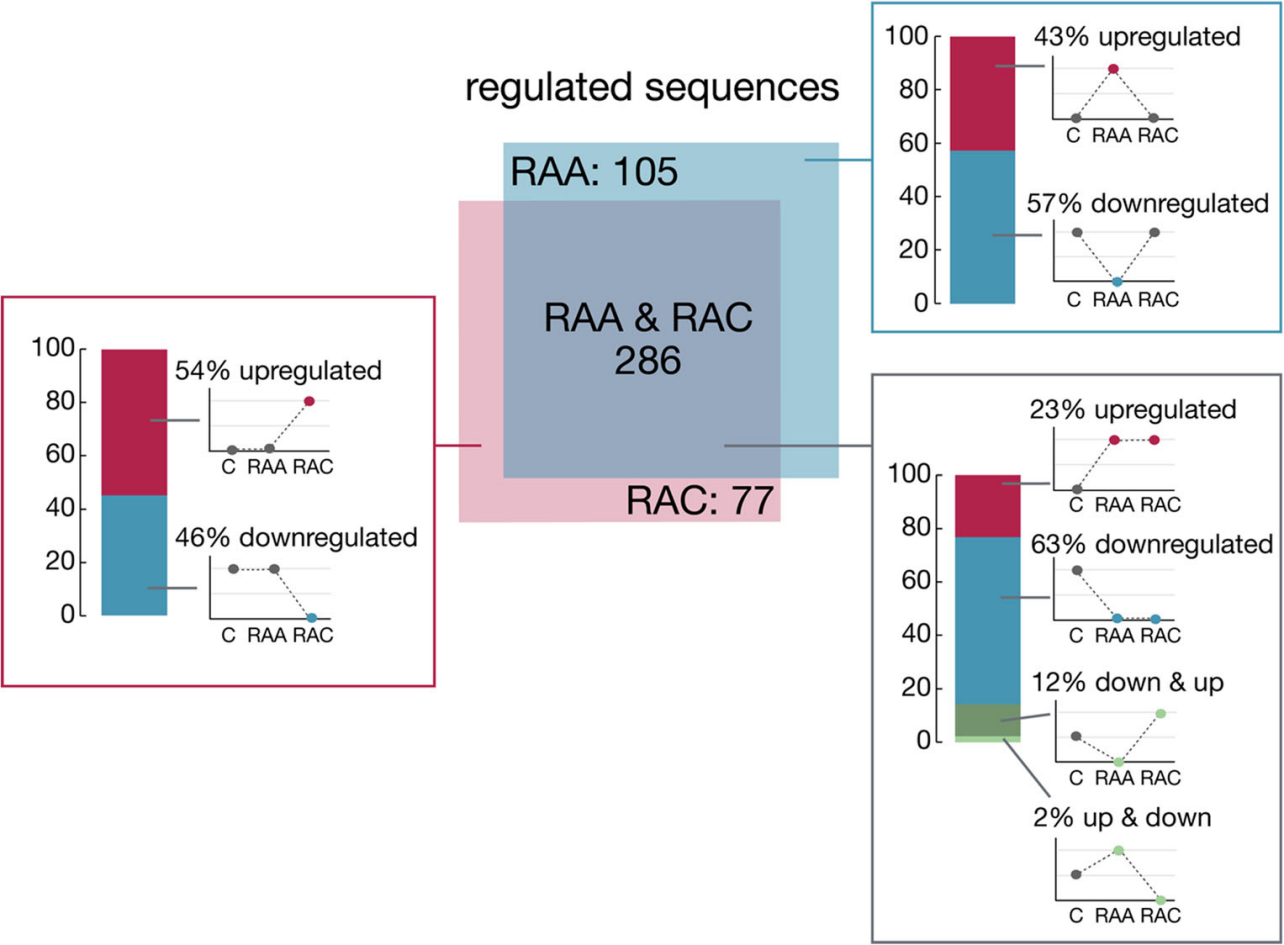
Timepoint dependence and direction of sRNA expression changes after IA rupture. The diagram in the middle represents the number of small RNAs that were significantly differentially expressed in the tested conditions. A total of 105 sRNAs were differentially expressed only in the blood from RAA patients (acute phase, days; blue shape), 77 sRNAs were differentially expressed only in the blood from RAC patients (chronic phase, months; pink shape), and 286 sRNAs were changed at both timepoints. Bar graphs adjacent to each part of the Venn diagram indicate the direction of sRNA transcript-level changes (blue, percentage of downregulated transcripts; red, percentage of upregulated transcripts; green, percentage of transcripts with a mixed expression profile). Small graphs to the right of each bar illustrate patterns of alterations in expression

The first goal of the analysis was to investigate classwise sRNA expression profiles of both the acute and chronic phases after IA rupture, as well as effects that were sustained between both timepoints. To identify sRNAs that were specifically regulated at each analyzed timepoint, sRNAs were considered regulated if they met the fold-change > 1.2 condition in one or both comparisons (Supplementary Table S1). We identified 105 sRNAs that were regulated in the acute phase and 77 sRNAs that were regulated in the chronic phase in comparison with controls. A total of 286 sRNAs were differentially expressed in both experimental groups compared with the control, suggesting that most of the investigated sRNAs underwent prolonged changes in their abundance in blood after IA rupture (Fig. 1).

To further elucidate sRNA regulation by IA rupture, we investigated the direction of change of sRNA expression in the acute and remote SAH period. Among the sRNAs regulated in the acute phase or in both, most (60 downregulated vs. 45 upregulated and 210 downregulated vs. 76 upregulated, respectively) were downregulated, whereas in the chronic phase-regulated transcripts, most (42 upregulated vs. 35 downregulated) were upregulated. Eighty-one transcripts showed a mixed pattern of expression, where 41 were downregulated in the acute phase and upregulated in the chronic phase, while 7 were upregulated in the acute phase and downregulated in the chronic phase (Fig. 1, Supplementary Table S1).

### Analysis of class composition of the regulated sRNAs

**Fig. 2:**
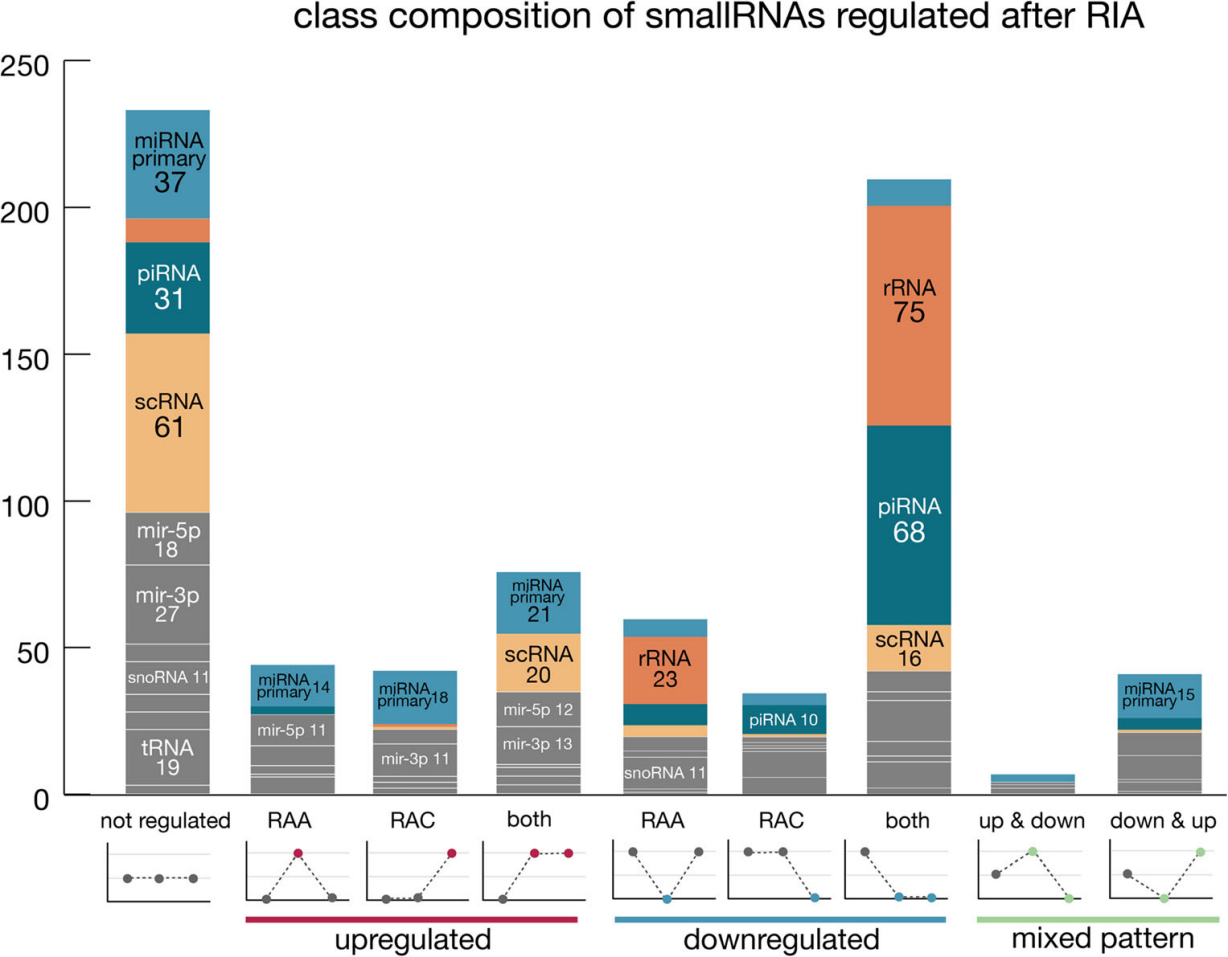
Class composition of the sRNAs regulated in response to aneurysm rupture. The height of each bar (*y*-axis) represents the number of sRNAs, with the bar sections corresponding to the number of sRNAs in each class. The class name and sRNA number are indicated for classes with 10 or more members in a given condition. The most abundant classes are additionally color-coded: dark blue: piRNA, light blue: primary miRNA, dark orange: rRNA, light orange: scRNA. Leftmost bar—a set of nonregulated sRNAs with their class composition. Subsequent bars show the composition of sRNA classes among the upregulated and downregulated transcripts at acute, chronic, and both time points. The rightmost two bars present the class composition of sRNA groups with mixed expression patterns. Details of numbers of sRNA sequences of each class and their regulation patterns are available in Supplementary Table S2

We next examined the number of regulated sRNAs of each RNA type. To investigate the specificity of the regulation patterns described, we compared the abundance of each sRNA class with a group of randomly selected, nonregulated sRNAs that included a similar number of sequences as the largest regulated group (*n* = 233, Fig. 2, Supplementary Table S2).

In general, the class composition of nonregulated sRNAs was clearly distinct from that of downregulated and upregulated sRNAs. Overall, miRNAs, rRNAs, piRNAs, and scRNAs were the most abundant classes, but their numbers differed between experimental groups. In the case of miRNAs, the number of transcripts was highest within the nonregulated group (*n* = 37 vs. *n* = 3–18 in other groups). Among the regulated transcripts, sRNAs downregulated in both SAH phases showed a radically different class composition compared with all the other groups, with rRNA and piRNA sequences being overrepresented (rRNA *n* = 75 vs. *n* = 0–23 in other groups, piRNA *n* = 68 vs. *n* = 0–31 in other groups). In summary, the class composition analysis showed a specific pattern of sRNA expression regulation, particularly among the downregulated transcripts.

### Analysis of sequence conservation of the identified sRNAs

Next, we analyzed how conserved the sequences of the regulated sRNAs are between the human genome and 99 other vertebrate genomes. To this end, we used available phastCons scores [11]. First, we investigated whether sRNA classes are differentially conserved among one another and between nonregulated and regulated sRNAs. This analysis was performed by comparing the 516 regulated sRNAs with a list of 457 nonregulated sRNAs (Supplementary Fig. S1). This analysis revealed that conservation among sRNA sequences is largely class-specific, with tRF3, tRF5, and scRNAs being the least conserved and tRNAs, piRNAs, and mir-5p having the highest numbers of conserved sequences. Out of the highly conserved classes, the piRNAs were overrepresented in the downregulated sRNAs in both the acute and chronic groups.

### Binding patterns of regulatory factors to genomic positions of regulated sRNAs

We searched for overrepresented transcription factor binding sites in genomic regions encoding the regulated sRNAs. In general, sRNA sequences exhibit strong ChIP-seq signals from multiple transcriptional factors, possibly due to the high transcriptional activity of these loci.

**Fig. 3:**
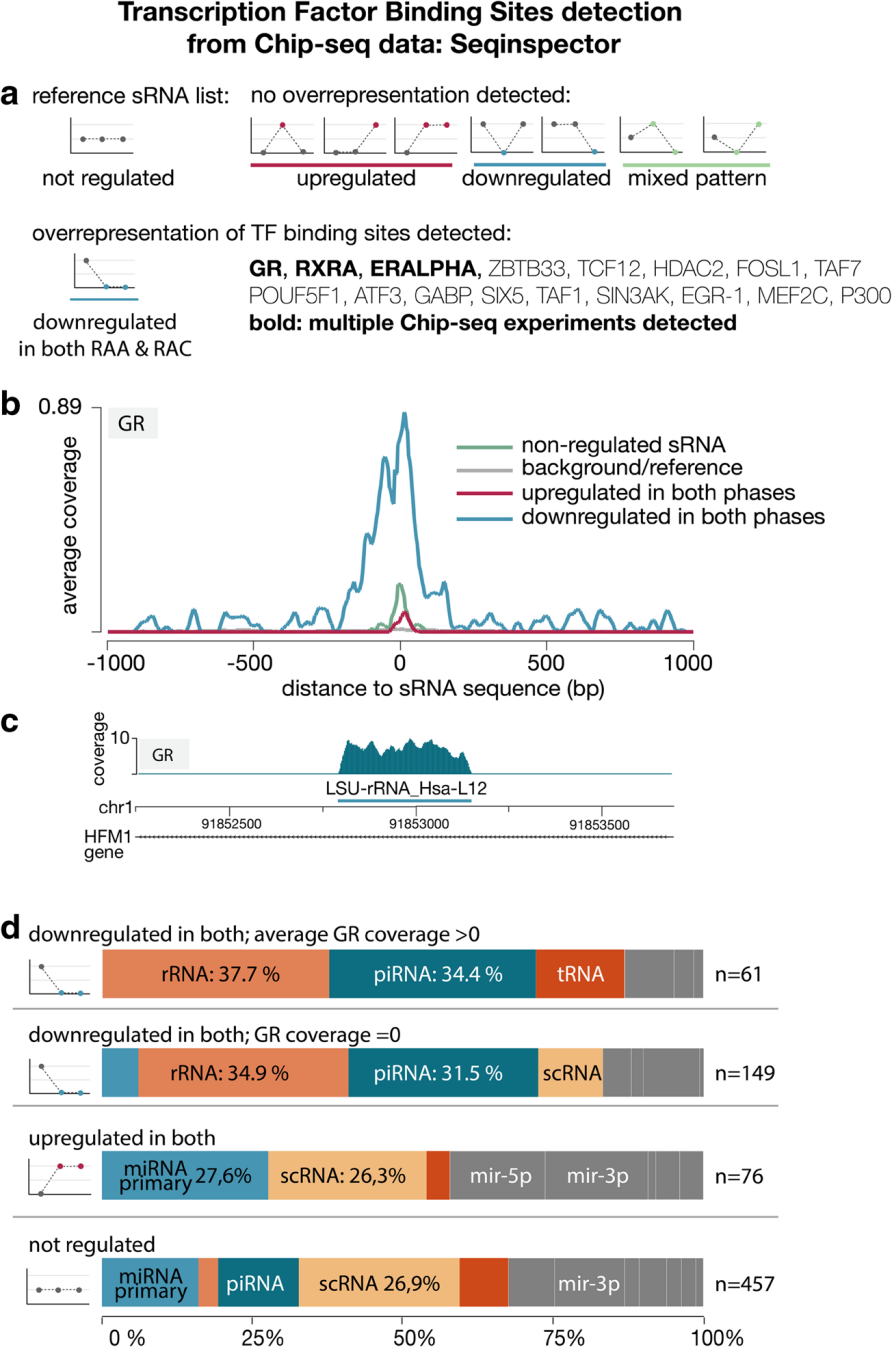
Overrepresentation of transcription factor (TF) binding sites in sRNAs downregulated after IA rupture with glucocorticoid receptor (GR) as an example. **a** Overrepresentation of TF binding sites was detected only in the sRNAs that showed downregulation in both phases. Nonregulated sRNAs were used as references. **b** Histogram of averaged ChIP-seq signal of downregulated sRNA sequences for GR (blue line). Upregulated sRNA sequences (red line), nonregulated sRNA sequences (green line, reference), and a genome background (gray line) are shown for comparison. **c** GR ChIP-seq signal for LSU-rRNA_Hsa-L12 genome location is shown as an example. The GR binding peak corresponds well with the rRNA sequence. **d** Class composition (in percentage) of downregulated sRNA sequences with detected representation of GR binding sites (uppermost panel, *n* = 61). This composition was compared with the class composition of downregulated sRNAs without GR binding sites detected (second panel, *n* = 149). Two bottom panels show the class composition of upregulated and nonregulated sRNAs. Color-coding as in Fig. 2, with the darkest orange added for tRNAs. The classes with > 20% abundance percentage are indicated

We compared the lists of upregulated and downregulated sRNAs in either acute, chronic, or both phases, with a list of nonregulated sRNAs serving as a reference. The regions expressing sRNAs downregulated in peripheral blood cells of patients after IA rupture (both acute and remote period) exhibit a significant overrepresentation of ChIP-seq signals for a set of transcription factors (TFs) (Supplementary Table S3). Among these TFs, overrepresentation was detected in more than one experiment for three factors (Fig. 3a): glucocorticoid receptor (GR), retinoid x receptor alpha (RXRA), and estrogen receptor alpha (ERALPHA). No overrepresentation of TF binding sites was detected for any other group of regulated sRNA sequences.

A histogram of GR ChIP-seq average coverage is shown in Fig. 3b. The peak signal is concentrated near the start of the sRNA sequence. An example ChIP-seq signal of an sRNA (large ribosomal subunit rRNA) with the highest GR signal is shown in Fig. 3c.

Classwise analysis indicated that the tRNA (particularly loci with tRNA-His-GTG-1) is the sRNA subtype with enriched representation of GR-binding in its vicinity (comparison of class composition of sRNAs downregulated in both phases that have vs. those that do not have GR ChIP-seq signal coverage, chi-square 59.77, *p* value < 0.0001). Many rRNAs (5S, SSU, and LSU) and piRNAs (including piR-44984, piR-36233, piR-31080, piR-48966, piR-48966, piR-57947, and piR-36329) also showed GR binding-site overrepresentation (Fig. 3d). A similar pattern was observed for the other TFs (Supplementary Fig. S2).

## Discussion

To the best of our knowledge, this report is the first to describe the effects of IA rupture on sRNA expression in peripheral blood cells. Transcriptome analysis revealed that alterations in the expression levels of sRNAs were present both in the acute and remote periods after SAH. Although the direction of observed changes was generally preserved, for a small subset of sRNAs, the directionality differed between phases. Since our knowledge on the functionality of particular RNA molecules is notably scarce, interpreting the obtained results remains difficult. Notably, the downregulated transcripts were predominantly rRNAs and piRNAs.

At the cellular level, stress leads to alteration of cell metabolism and reorganization of nuclear architecture, reflecting the simultaneous inhibition and activation of specific nuclear pathways. Since ribosome biosynthesis is a highly energy-consuming process, one strategy to preserve energy homeostasis is the attenuation of ribosomal biogenesis and rRNA synthesis. This can be achieved by downregulating rDNA transcription, pre-RNA processing, and epigenetic regulation by adjusting the number of active genes. The size of the nucleolus, which plays a crucial role in ribosome biogenesis, is positively correlated with the rRNA synthesis rate, which depends on cell growth and metabolism [12–14]. Thus, we can assume that the observed downregulation of rRNA is a response of peripheral blood cells to stress caused by IA rupture. This finding corresponds with lymphopenia present in SAH patients. Although we observed a simultaneous increase in monocyte count, considering the absolute numbers of cell classes, it seems that the drop in lymphocyte count is more prominent. Moreover, we have previously reported in the acute phase of SAH a downregulation of *HEATR1* (HEAT repeat-containing protein 1) mRNA. HEATR1 protein is involved in the regulation of ribosomal biogenesis, and its downregulation causes cell cycle arrest [4].

Another type of downregulated sRNA is piRNAs. These RNAs are predominantly recognized as molecules responsible for the regulation of the activity of transposons, which constitute approximately 45% of the human genome. Since transposons can mobilize in the genome and cause genomic instability, piRNAs are recognized as molecules crucial for genome stability and the prevention of mutagenesis. Moreover, piRNAs are involved in epigenetic control via DNA methylation and histone modification [6, 15]. This control was demonstrated not only in germline cells but also in somatic and cancer cells [16, 17]. The significance of the observed alterations in piRNA expression and their pathophysiological role after SAH remains to be elucidated. Transposons are implicated in the immune response in multiple sclerosis and other autoimmune conditions [18]. Rajan et al. showed altered piRNA expression in the heart during hypertrophy induction in a rat model. These researchers also found differences in the abundance levels of circulating piRNA in the serum of patients with myocardial infarction, suggesting the importance of piRNA-mediated mechanisms in various pathophysiological conditions affecting the cardiovascular system [19]. Dysregulation of piRNA expression has been shown in many different human malignancies [20]. Additionally, in a rat cerebral ischemia model, alterations in piRNA expression were demonstrated in the ischemic cortex [21].

Another interesting observation is differences in conservation scores among different classes of regulated sRNAs, particularly the low conservation score of tRFs compared with the high conservation score of tRNAs. tRNAs together with rRNAs are housekeeping RNAs. These RNAs are responsible for the supplying amino acids to ribosomes and other biochemical pathways and regulating cell apoptosis by cytochrome binding, and they are highly conserved in evolution [22]. Interestingly, the largest tRNA gene cluster is located in the major histocompatibility complex (MHC) region, which is crucial in adaptive and innate immunity [23]. tRFs are derivates of tRNAs and are known to accumulate in response to cellular stress. tRFs play an important regulatory role in gene expression (at the level of transcription and translation), apoptosis, and cell survival. There is a growing body of evidence indicating that tRF generation is not a random process, and their function may vary between cell types [24–26]. For instance, in monocytes, *tRF-5030c* via PIWI protein interaction was shown to cause histone H3K9 methylation [27]. Veneziano et al. found significant dysregulation of tRF expression in human chronic lymphocytic leukemia, suggesting that this class of molecule could at least serve as a disease biomarker [28]. Thus, one can speculate that alterations in tRF expression are specific for the systemic response to SAH.

Analysis of TFBSs among regulated sRNAs revealed significant overrepresentation of ChIP-seq signals for a range of transcription factors. The top 3 overrepresented TFBSs, namely, GR, ERALPHA, and RXRA, belong to the superfamily of nuclear receptors, and they are all overrepresented among downregulated sRNA classes.

GR is expressed in nearly all vertebrate cells. GR is involved in the regulation of thousands of genes and participates in different aspects of development, metabolism, stress response, and other processes. GR can interact with DNA directly or via proteins. Additionally, there are many coregulators of these interactions, and most GR target genes are cell type-specific. As a result, the final response to GR stimulation is context-specific and distinct from the cell type [29]. Glucocorticoids (GCs) are endogenous agonists of GR. GCs are known to exert potent regulatory effects on the immune system and inflammatory response. GCs are negative regulators of thymopoiesis, possess lympholytic properties, affect T cell activation and polarization (promoting Th2 and Treg), and influence monocyte function [30, 31]. Increases in circulating GC levels caused by the activation of the hypothalamic-pituitary-adrenal axis (HPAA) were shown in cerebral injuries. A dysregulation of HPAA in SAH patients with an abnormal diurnal cortisol profile was reported [32, 33]. In an ischemic stroke model, Courties et al. demonstrated that activation of HPAA with high systemic levels of GCs affected lymphopoiesis by inducing GR-dependent apoptosis in thymic T cells and in B-cell progenitors [34]. Similar effects of traumatic brain injury on T cells were reported [35]. Our results suggest that GR may also play a regulatory role by interacting with some sRNAs.

Additionally, the retinoid X receptor (RXR) plays a role in the regulation of the immune system. The RXR agonists decrease apoptosis of T cells and promote Th2 polarization, whereas disruption of the RXRA gene in mice resulted in attenuation of T-cell proliferation and promotion of their apoptosis [36]. In addition, disruption of RXR function in macrophages impaired phagocytosis, and RXR activation increased the expression of TGFbeta1 [37]. In rodent models of SAH and brain ischemia, activation of RXR played a protective role in damping neuroinflammation [38–40]. In human carotid atherosclerotic plaques, decreased expression of RXR in macrophages and smooth muscle cells was associated with the severity of atherosclerosis [41].

It is widely recognized that estrogen receptors (ERs), including ERALPHA, have prominent effects on immune function in both innate and adaptive immune responses. ERALPHA is expressed in a variety of immune cells and plays an important role in the modulation of cytokine production, cytokine receptor expression, and the activation of effector cells. In general, the stimulation of ERALPHA exerts anti-inflammatory effects. The significance of ERALPHA-mediated signaling in autoimmune disorders is well recognized [42]. Estrogens are known to play a neuroprotective role in stroke, primarily attenuating the inflammatory response [43–45]. The current results indicate that the immunity-related function of ERALPHA can be mediated by interactions with sRNAs.

Our study has a number of limitations. One limitation is the limited sample size. However, the numbers of patients investigated here (*n* = 59) and in other studies focused on miRNAs are largely comparable, with other studies including from 24 up to 183 participants [46–49]. Furthermore, presented results have not been validated in an independent cohort, thus should be considered proof-of-concept. Further studies are needed to evaluate the reproducibility of observed chronic dysregulation of sRNA levels in IA rupture. Next, the interpretation of the obtained results is speculative to a certain extent. The current knowledge about the role played by sRNAs in humans remains highly limited. In addition, transcripts often exist at multiple less-annotated loci and without full functional description of the products. Overrepresentation of TFBSs highly transcribed loci has been previously reported and remains a plausible explanation of the observed phenomenon [50]. Interestingly, a prominent overrepresentation of GR among TFBSs in regulated sRNAs corresponds with our understanding of the role played by GCs in acute stress and immune/inflammatory reactions.

In the light of discussed limitations, presented results should be considered a proof-of-concept study. Our main observations are the differences in sRNA expression between the experimental and control groups and the result that the downregulation of many sRNA transcripts persists in the chronic phase after IA rupture. These should further be validated in a separate, preferably larger, cohort of patients. If the results are further validated, there is a translational clinical potential in the presented observations. Namely, levels of sequences of various sRNA classes (such as rRNA, tRNA, miRNA) could serve as potential IA biomarkers; however, further studies are needed. The interpretation of the observed differences may change when better genome annotations and functional descriptions of the small RNAs are available.

In conclusion, the results of this study provide novel insights into sRNA regulation in relation to the systemic inflammatory response after IA rupture. Specifically, we found that SAH is associated with the downregulation of sRNAs that are involved in the regulation of transcription and translation processes. These alterations in the expression levels of some sRNA classes are probably dependent on the activity of the nuclear receptors GR, ERALPHA, and RXRA.

## Electronic supplementary material

ESM 1 (XLSX 69 kb)

ESM 2 (PDF 512 kb)

ESM 3 (XLSX 13 kb)

ESM 4 (XLSX 11 kb)
