# Supplementary material for: Opposite regulation of piRNAs, rRNAs and miRNAs in the blood after subarachnoid hemorrhage
Source: J Mol Med (Berl). 2020 May 18;98(6):887–96. doi: 10.1007/s00109-020-01922-x (PMC7297814; doi:10.1007/s00109-020-01922-x)
Supplement: Supplementary file 2 — (PDF 512 kb) [file 109_2020_1922_MOESM2_ESM.pdf]

SUPPLEMENTARY MATERIAL

to "Opposite regulation of piRNAs, rRNAs and miRNAs in the blood after subarachnoid hemorrhage"

Journal of Molecular Medicine

Michal Korostynski[1], Marcin Piechota[1], Rafal Morga[2], Dzesika Hoinkis[3], Slawomir Golda[1], Magdalena Zygmunt[1], Tomasz Dziedzic[4], Marek Moskala[2], Agnieszka Slowik[4], Joanna Pera[4]

[1]Department of Neurosurgery and Neurotraumatology, Faculty of Medicine, Jagiellonian University Medical College, ul. Botaniczna 3, 31-503 Krakow, Poland

[2]Department of Molecular Neuropharmacology, Institute of Pharmacology, Polish Academy of Sciences, ul. Smetna 12, 31-343 Krakow, Poland

[3]Intelliseq sp. z o.o. , ul.. Chabrowa 12/3, 31-335 Krakow, Poland

[4]Department of Neurology, Faculty of Medicine, Jagiellonian University Medical College, ul. Botaniczna 3, 31-503 Krakow, Poland

Corresponding author:

Joanna Pera

e-mail: joanna.pera@uj.edu.pl, pera@su.krakow.pl

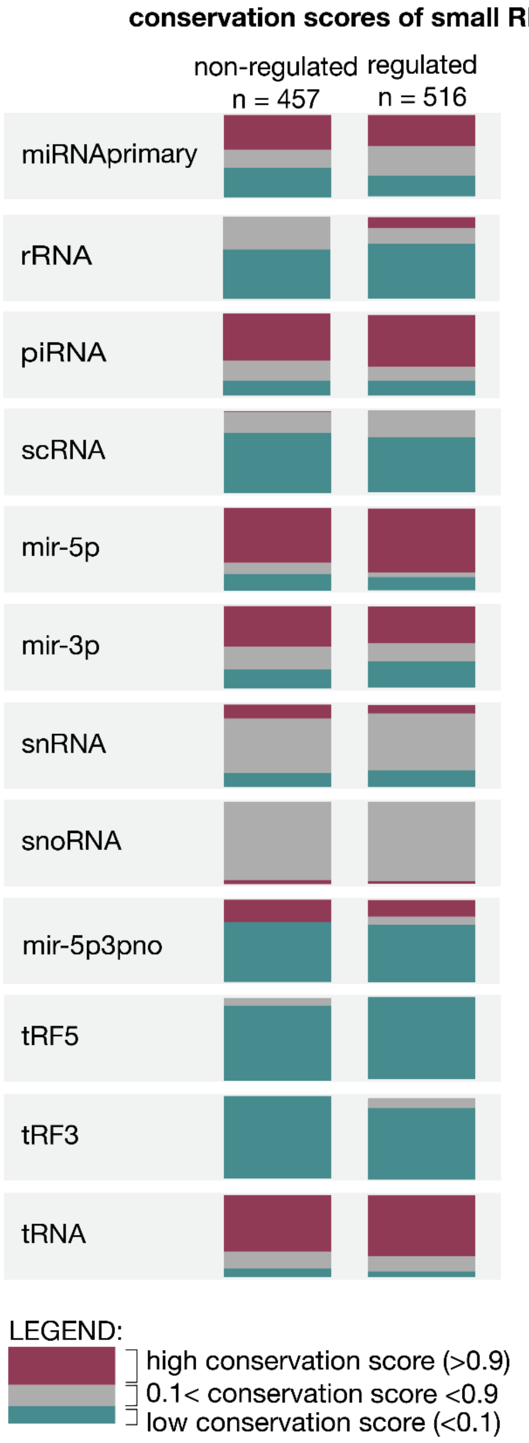

Supplementary Fig. S1 Percentage of variable and conserved sequences in nonregulated and regulated sRNAs per class in each group

The height of each section represents the percentage a given group has among each class. Variable (blue sections) sRNAs are those with conservation scores <0.1, conserved (red sections) sRNAs are those with conservation scores >0.9, and sRNAs with conservation values in between have been marked gray (0.1< score <0.9). Chi-square statistics were performed and did not show any significant changes in conservation between groups within class.

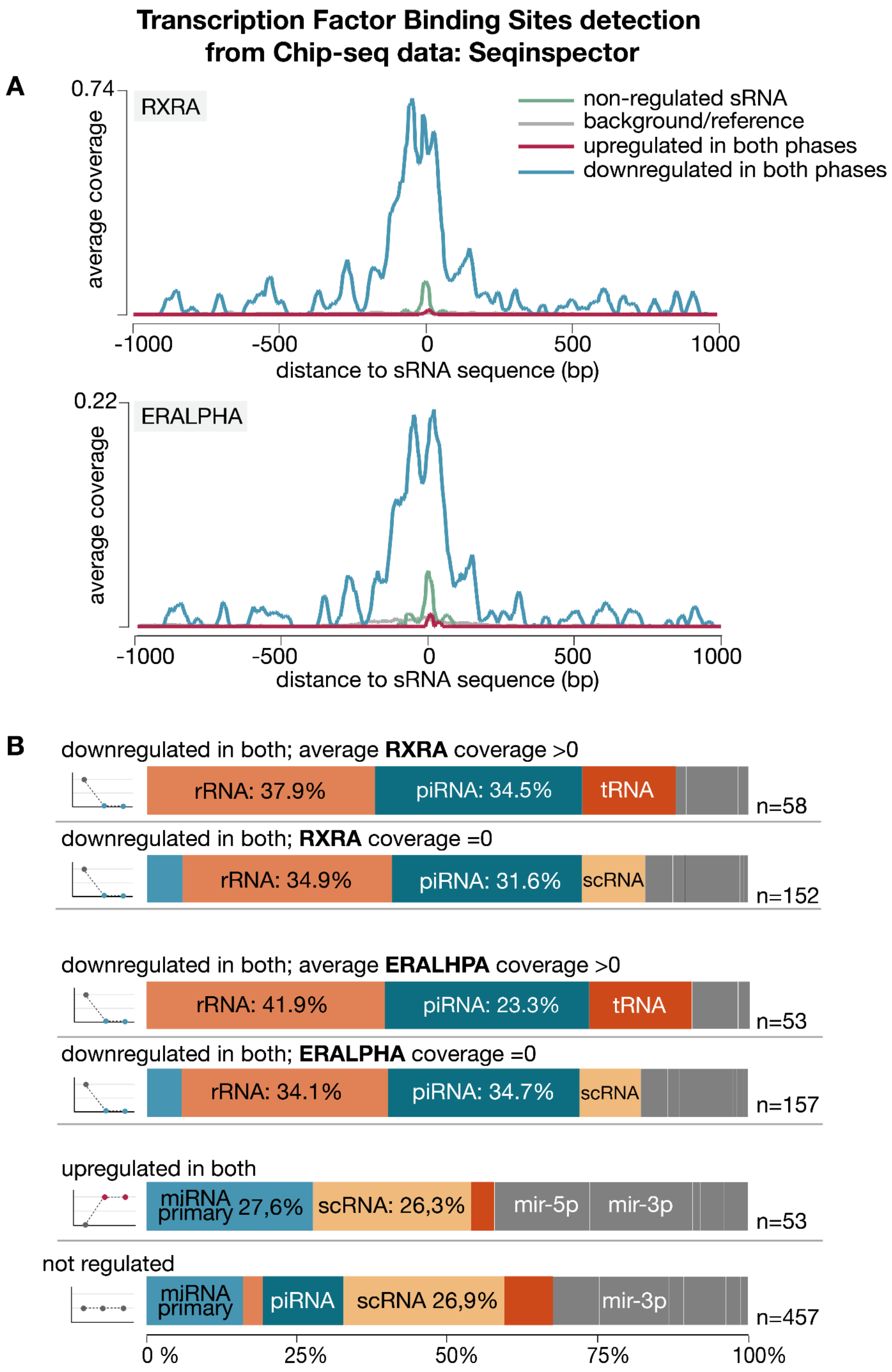

Transcription Factor Binding Sites detection
from Chip-seq data: Seqinspector
A
0.74
RXRA
average coverage
non-regulated sRNA
background/reference
upregulated in both phases
downregulated in both phases
-1000
-500
0
500
1000
distance to sRNA sequence (bp)
0.22
ERALPHA
average coverage
-1000
-500
0
500
1000
distance to sRNA sequence (bp)
B
downregulated in both; average RXRA coverage >0
rRNA: 37.9%
piRNA: 34.5%
tRNA
n=58
downregulated in both; RXRA coverage =0
rRNA: 34.9%
piRNA: 31.6%
scRNA
n=152
downregulated in both; average ERALHPA coverage >0
rRNA: 41.9%
piRNA: 23.3%
tRNA
n=53
downregulated in both; ERALPHA coverage =0
rRNA: 34.1%
piRNA: 34.7%
scRNA
n=157
upregulated in both
miRNA primary 27,6%
scRNA: 26,3%
mir-5p
mir-3p
n=53
not regulated
miRNA primary
piRNA
scRNA 26,9%
mir-3p
n=457
0 %
25%
50%
75%
100%

Supplementary Fig. S2 Overrepresentation of retinoid X receptor alpha (RXRA) and estrogen receptor alpha (ERALPHA) binding sites in sRNAs downregulated after IA rupture A.
Histograms of averaged ChIP-seq signal of sRNA sequences for RXRA (upper histogram) and ERALPHA (lower histogram). Downregulated sRNA sequences – blue lines, upregulated sRNA sequences - red lines, nonregulated sRNA sequence - green lines (reference) and a genome background (gray lines) are shown for comparison. B. Class composition (in percentage) of sRNA sequences with or without detected representation of RXRA/ERALPHA binding sites.

SUPPLEMENTARY TABLES – DESCRIPTION

Supplementary Tables S1, S2, S3 are available in uploaded Excel Files.

Supplementary Table S1
Differentially expressed sRNAs
Localization, class, ID and expression of sRNAs from subjects in the acute phase of IA rupture (RAA), chronic phase of IA rupture (RAC) and control subjects (C) analyzed using small RNA-seq. The table presents a list of 516 sRNAs with significant differences in terms of RNA abundance levels between the experimental groups. The regulated sRNAs are listed with their mean abundance level in each group (mean_C, mean_RAA and mean_RAC columns), fold-change compared to the control (FC_RAA, FC_RAC) and the FDR value obtained from edgeR. The last three columns describe the change direction. "C.vs. RAA: DOWN" means that the particular sequence has a lower mean abundance in the RAA group than in the C group.

Supplementary Table S2
Changes in sRNA levels based on classes
This table enumerates sRNAs belonging to each of the studied classes and their regulation patterns. This table supplements Figure 2.

Supplementary Table S3
Statistics for transcription factor ChIP-seq signal from sequences downregulated in both RAA and RAC
Statistics were obtained with the Seqinspector tool. A total of 210 sequences from the "both downregulated" category were compared with a list of 454 sRNA sequences that were not regulated by IA rupture and had similar expression levels. Query – signal height from tested (210) list, Background – signal from nonregulated sRNAs. Fold diff – difference between the query and the background. P value – p-value of test for difference in ChIP-seq signal intensity. Bonferroni – corrected p-value.
